# Fibroblasts Isolated from Human Middle Turbinate Mucosa Cause Neural Progenitor Cells to Differentiate into Glial Lineage Cells

**DOI:** 10.1371/journal.pone.0076926

**Published:** 2013-10-21

**Authors:** Xingjia Wu, William E. Bolger, Juanita J. Anders

**Affiliations:** 1 Department of Anatomy, Physiology and Genetics, Uniformed Services University of the Health Sciences, Bethesda, Maryland, United States of America; 2 Department of Otorhinolaryngology, Mayo Clinic Medical School, Jacksonville, Florida, United States of America; Oregon Health & Science University, United States of America

## Abstract

Transplantation of olfactory ensheathing cells (OECs) is a potential therapy for repair of spinal cord injury (SCI). Autologous transplantation of OECs has been reported in clinical trials. However, it is still controversial whether purified OECs or olfactory mucosa containing OECs, fibroblasts and other cells should be used for transplantation. OECs and fibroblasts were isolated from olfactory mucosa of the middle turbinate from seven patients. The percentage of OECs with p75^NTR+^ and GFAP^+^ ranged from 9.2% to 73.2%. Fibroblasts were purified and co-cultured with normal human neural progenitors (NHNPs). Based on immunocytochemical labeling, NHNPs were induced into glial lineage cells when they were co-cultured with the mucosal fibroblasts. These results demonstrate that OECs can be isolated from the mucosa of the middle turbinate bone as well as from the dorsal nasal septum and superior turbinates, which are the typical sites for harvesting OECs. Transplantation of olfactory mucosa containing fibroblasts into the central nervous system (CNS) needs to be further investigated before translation to clinical application.

## Introduction

Spinal cord injury (SCI), whether by mechanical destruction or disease, causes complete or partial loss of sensation and motor function due to the interruption of signal conduction along the severed axon tracts. Due to limited axonal regeneration within the central nervous system (CNS), many potential therapies have been investigated for repair of SCI. Cell-based therapy is a widely studied repair strategy that involves transplantation of one or several cell types into injured spinal cords. Transplantation of a variety of cells has been investigated for their therapeutic efficacy for SCI repair including Schwann cells, olfactory ensheathing cells (OECs), neural stem/progenitor cells, and bone marrow derived mesenchymal or hematopoietic stem cells (see reviews, [1], [2]). OECs, a type of supportive glia, ensheath olfactory neuron axons between the central and peripheral nervous systems. This cell type supports the continual regrowth of olfactory neuron axons throughout life. Transplantation of OECs has been reported to support axonal regeneration and functional recovery in both transection and contusion injury animal models [3], [4], [5], [6], [7]. This promising cell therapy has triggered efforts to convert animal research into worldwide clinical trials [8], [9], [10], [11], [12].

OECs can be harvested from the nerve fiber layer of the olfactory bulb or the nasal olfactory mucosa. When considering the source of OECs for human transplantation, harvesting OECs from nasal mucosa has advantages over harvesting OECs from the olfactory bulb. OECs from olfactory mucosa are located in the nasal cavity and accessible by minimally invasive endoscopic sinonasal surgery [13] and partial removal of the olfactory mucosa does not significantly alter olfactory function [14]. Within the human nasal cavity, OECs are present in the mucosa of the superior turbinate, nasal septum and middle turbinate [15]. The nasal septum and caudal portions of the superior turbinate contain the highest number of OECs [16]. Although clinical trials have demonstrated the feasibility and safety of OEC transplantation, it is controversial whether purified mucosal OECs, mixed cell cultures from olfactory mucosa, or pieces of whole mucosa should be used for autologous transplantation in humans. It has been suggested that many cell types from human olfactory mucosa may contribute to spinal cord repair [17]. However, the characteristics of each cell type from human mucosa and how they will react within the human spinal cord after transplantation have not been established.

Fibroblasts from meninges are a main contributor to fibrous scar formation after penetrating SCI [18]. When meningeal fibroblasts interface with reactive astrocytes, basal lamina is deposited and a glial-fibrotic scar is formed to reestablish CNS homeostasis [18]. This scar formation creates a barrier not just to fibroblast invasion, but also to axonal regeneration through the injured area. Transplantation of fibroblasts after either transection or contusion models of SCI resulted in limited or no functional recovery [19], [20]. A large ED1 positive macrophage response was also found around the transplantation site after fibroblast injection [21]. By contrast, grafts of small pieces from the outer layer of olfactory bulb, containing both OECs and olfactory nerve fibroblasts, restored ipsilateral breathing rhythm and improved climbing in a rat hemisection model of the upper cervical spinal cord [22]. It has been suggested that olfactory nerve fibroblasts are as essential to spinal tract repair as they are to olfactory nerve repair [23]. Whether implants should contain fibroblasts is still controversial and needs further study.

The objective of this study was to identify the potential effects of cell/tissue transplantation that includes fibroblasts by co-culturing fibroblasts isolated from the lamina propria with normal human neural progenitors (NHNPs). The ability to culture human OECs from nasal mucosa of the middle turbinate region was also described.

## Materials and Methods

### Patients and Surgery

Human sinonasal tissue, especially middle turbinate tissue was resected as part of a therapeutic procedure to treat sinusitis or as part of a surgical approach to deeper sinonasal structures. This turbinate tissue did not contain important clinical or histologic information for the patient; therefore, patients could consent to allow us to use this tissue for this research project. Consent forms were signed and obtained from 7 patients. The consenting procedure and consent form were approved by the ethics committee before use. Human research participation in this study was in accordance with the Uniformed Services University (USU) Institutional Review Board (IRB) (G170OA) and Walter Reed Army Medical Center (WRAMC) IRB (03-32018) approved protocol.

Middle turbinate tissue was removed in the following fashion. Under endoscopic visualization, a pair of small nasal scissors was used to cut the mid-portion of the lamellar aspect of the middle turbinate, starting anteriorly, just above the lower bulbous aspect of the turbinate, cutting posteriorly into the superior meatus. The superiorly based vertical lamella of the turbinate was preserved. The turbinate was released from the lateral nasal wall by transecting the horizontal aspect approximately 8 mm from its insertion into the lateral nasal wall in the region of the sphenopalatine foramen. The freed turbinate tissue was removed from the nasal cavity with a straight forceps.

### Culture of Human Fibroblasts and OECs from Nasal Mucosa of Middle Turbinate

Once removed, tissues were placed immediately in ice-cold Dulbecco’s minimum essential media (DMEM). Middle turbinate mucosa was separated from the underlying conchal bone and incubated in Dispase II solution (2.4 U/ml, Roche) at 37°C for 1 hour. Lamina propria was carefully separated from the epithelium and cut into small pieces. Pieces of lamina propria were incubated in Collagenase A (2.5 mg/ml, Roche) (37°C, 20 minutes) and mechanically dissociated. The enzyme activity was stopped by addition of 0.5 mM EDTA. After centrifugation (300 g×5 min), the cell pellet was resuspended in growth medium (GM, containing DMEM and Ham’s F-12 at a 1∶1 mixture with 10% fetal bovine serum, 1% glutamine, 2% penicillin-streptomycin, and 1% gentamicin) [24]. Cells were seeded in uncoated flasks/chamber slides for 18 hours (37°C, 5% CO_2_). The majority of cells attached to the slides during this incubation period were fibroblasts, which were used as the source of human fibroblasts in the co-culture experiment. The supernatant was then transferred to poly-L-lysine coated chamber slides for 36 hours and transferred again into another set of lysine-coated chamber slides. The slides were maintained in an incubator (37°C, 5% CO_2_) for 8 days or until the cells were near confluence. The media was changed every 3 days. All culture media above were purchased from Invitrogen and chemicals from Sigma, except where noted.

### Cell Quantitation for Purity Assessment

After culturing, slides were washed once with PBS and then fixed using 4% paraformaldehyde for 10 minutes at room temperature (RT). After immunocytochemistry, labeled cells were visualized with a Nikon (Tokyo, Japan) Labophot fluorescent microscope. Several spots (20×magnification lens, spot size: 0.38 mm^2^) from each slide were randomly chosen so that 50–100 cells were counted for each slide. Each tissue sample had duplicate slides so that a total of 100–200 cells were counted for each patient sample.

### Co-culture of Fibroblasts with Normal Human Neural Progenitors

NHNPs were obtained as cryopreserved neurospheres from primary cultures (Cambrex Bio Science Walkersville, Inc.). Cells were thawed and plated in neural progenitor maintenance medium (NPMM) for 24 hours according to protocols provided. For the co-culture experiments, NHNPs were plated on a semi-confluent cell layer of fibroblasts. The seeding density of neural progenitor cells was 25,000–50,000 cells/cm^2^. As a control, NHNPs were plated on chamber slides coated with 0.05% polyethylenimine (PEI) solution (Cambrex Bio Science Walkersville, Inc.). To compare the effect of the medium used on differentiation of NHNPs, co-culture was performed in either NPMM or GM. After 3 days of co-culture, cells were fixed and immunostained for GFAP and β-Tubulin III, as described below. Each culture condition was repeated at least twice.

### Immunocytochemistry

Cells were fixed using 4% paraformaldehyde. The following primary antibodies were used: mouse anti-human Thy1.1 (1∶1200, Serotec), rabbit anti-human basic fibroblast growth factor (FGF2, 1∶100, Santa Cruz Biotechnology), mouse anti-human Nestin (1∶200, Chemicon), mouse anti-human chondroitin sulfate proteoglycan (CSPG, 1∶100, Chemicon), mouse anti-human fibronectin (1∶100, Chemicon), rabbit anti-glial fibrillary acidic protein (GFAP, 1∶250, DAKO), mouse anti-human low affinity neurotrophin receptor p75 (p75^NTR^, 1∶750, USBiological), and mouse anti-β Tubulin III (anti-Tubulin, 1∶75, Sigma). Cells that were labeled for FGF2, Nestin, fibronectin, GFAP and β-Tubulin III were permeabelized by adding 0.1% Triton X-100 in the blocking solution (10% normal goat serum in phosphate buffered saline (PBS)). All primary antibodies were incubated for 1 hour at room temperature. Cy3-conjugated goat anti-mouse IgG (1∶500), Cy3-conjugated goat anti-rabbit IgG (1∶500) and fluorescein (FITC)-conjugated goat anti-rabbit IgG (1∶50) were used as secondary antibodies. All the secondary antibodies were purchased from Jackson ImmunoResearch Laboratories. Secondary antibodies were incubated for 30 minutes at RT. Double labeling with anti-p75^NTR^ and anti-GFAP or anti-Thy1.1 and anti-GFAP was described previously [24]. When double labeling with anti-GFAP and anti-β Tubulin III was performed, the two primary antibodies were used in a mixture followed by incubation with a mixture of the two related secondary antibodies. The cultures were washed with PBS after incubation of antibody. Nuclei were visualized by mounting slides using media with DAPI (Vector Laboratories). Negative controls were performed using the same procedure except the primary antibody was absent. No labeling was found in the negative control.

## Results

### Fibroblasts

**Figure 1:**
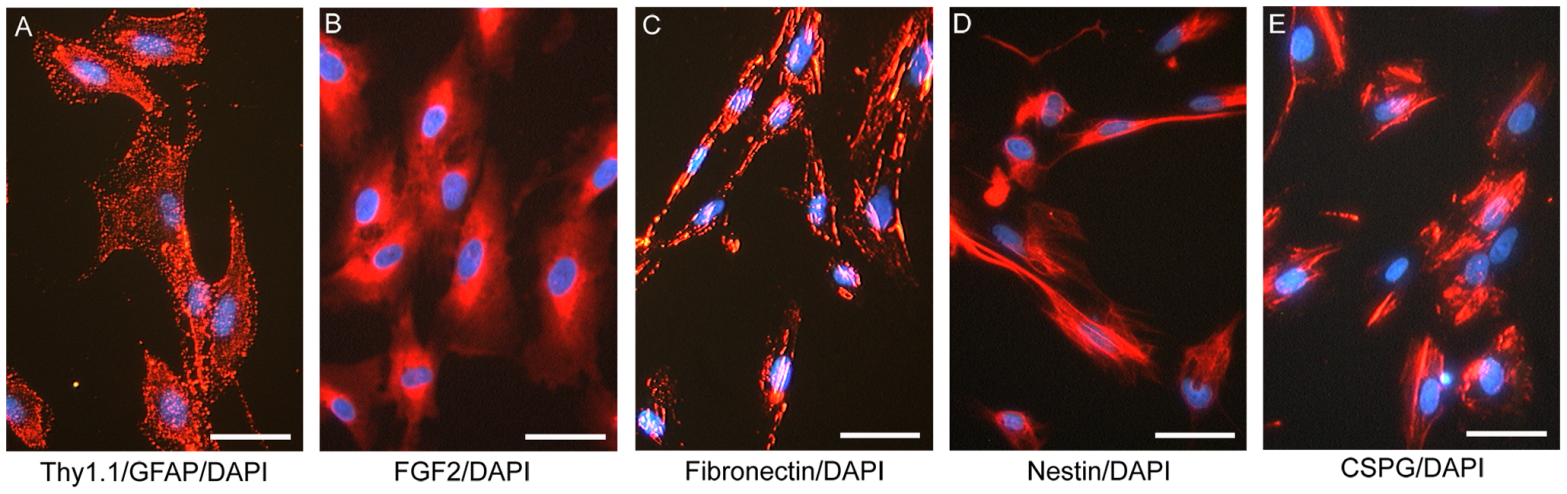
Immunocytochemistry of fibroblasts from human mucosa of middle turbinate. Immunocytochemistry of fibroblasts was performed using antibodies of Thy1.1 (fluorescently tagged red) and GFAP (green) with DAPI (blue) (A). GFAP labeling was negative in the fibroblasts from middle turbinate. Fibroblasts were labeled with FGF2 (B), Fibronectin (C), Nestin (D) and CSPG (E). Nuclei were visualized with DAPI (blue). Scale bars: 50 µm.

The majority of the cells isolated during the first step of the enrichment procedure were fibroblasts from the lamina propria (95.6% of total cells), identified by Thy1.1^+^ GFAP^−^(Fig. 1A). These fibroblasts also presented other antigenic markers typically exhibited by fibroblasts including FGF2 (Fig. 1B) and fibronectin (Fig. 1C). Furthermore, Nestin (Fig. 1D) and CSPG (Fig. 1E) were detected in these fibroblasts. Expression of Nestin has been reported in fibroblasts cultured from human olfactory mucosa [25], [26] and CSPG has been reported to be produced in the human embryonic skin fibroblasts [27], human lung fibroblasts [28] and periodontal fibroblasts [29], [30]. Greater than 95% of the cells were positive for Thy1.1, FGF2 and fibronectin. Cells positive for Nestin and CSPG were not counted.

### OECs from Human Nasal Mucosa in the Middle Turbinate

**Figure 2:**
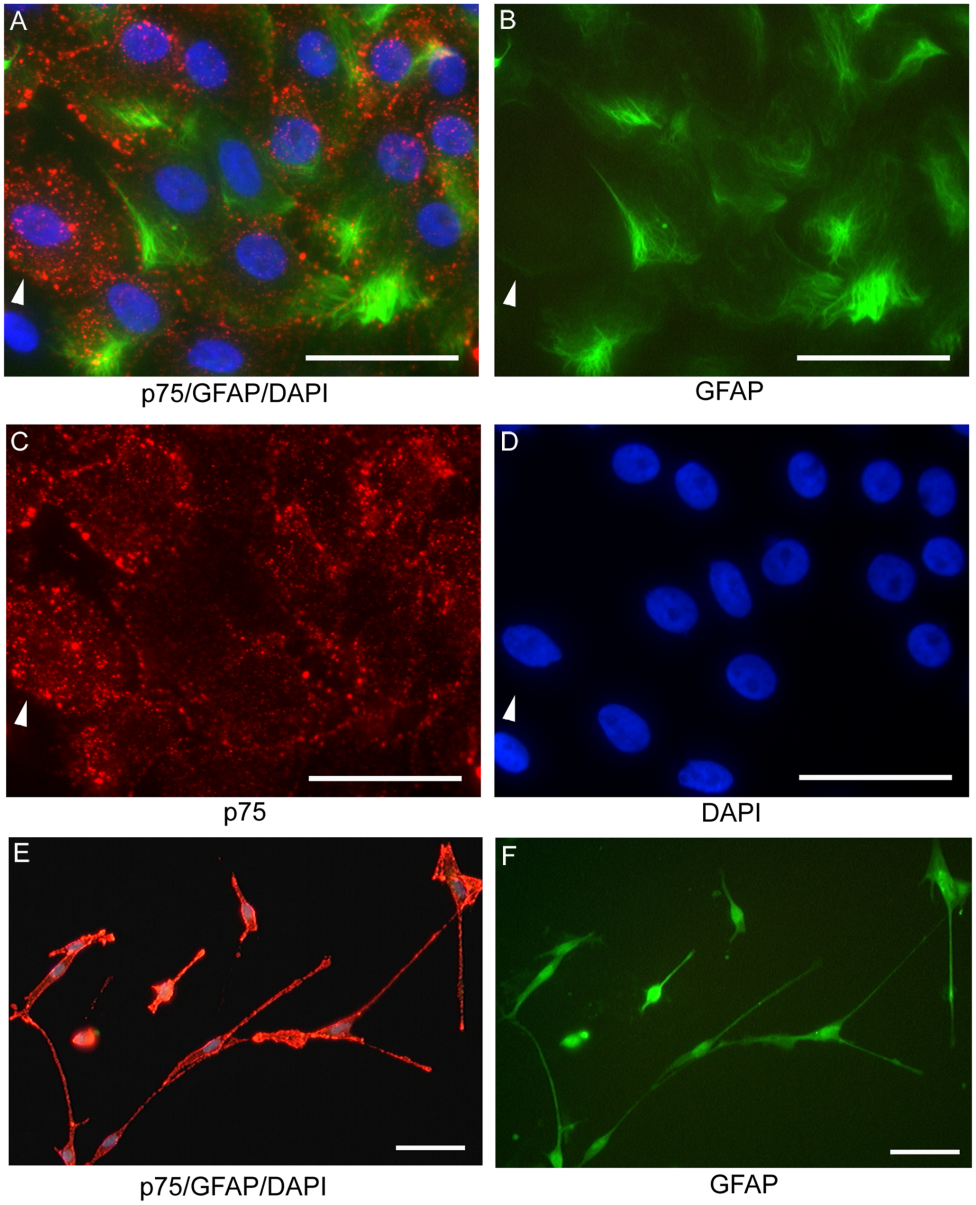
Culture of OECs from human mucosa of middle turbinate. Two different morphologies of OECs were found. Both types were both GFAP and p75^NTR^ positive labeled. One type (A–D) with filamentous GFAP labeling (B) and punctate p75^NTR^ labeling (C). Arrow head identifies one cell with single p75^NTR^ labeling. Cells labeled only with p75^NTR^ were not counted as OECs in this study. Another type (E, F) had intense p75^NTR^ labeling (red) and diffuse GFAP labeling (green). Scale bars: 50 µm.

Two morphologically distinct types of OECs identified by immunolabeling with GFAP and p75^NTR^ were found in the final step of the enrichment. One type (Fig. 2A–D) had a flat morphology with filamentous GFAP labeling (Fig. 2B) and punctate p75^NTR^ labeling (Fig. 2C). The other type had bipolar/multipolar morphology with intense p75^NTR^ labeling (Fig. 2E). Diffuse cytoplasmic GFAP labeling was present in this cell type (Fig. 2F).

**Figure 3:**
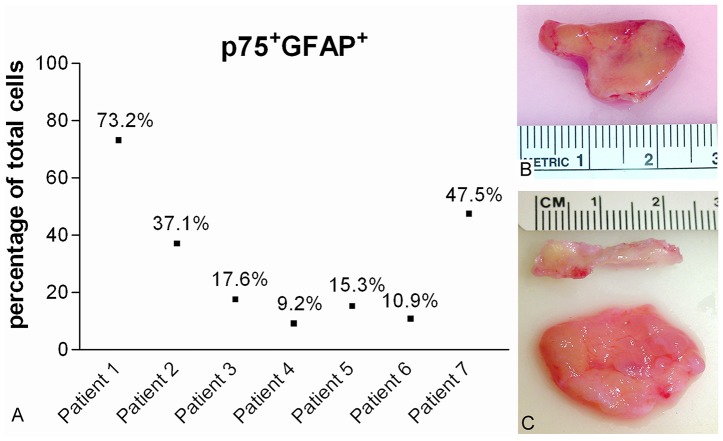
Percentage of OECs cultured from human middle turbinate mucosa. The percentage of p75^NTR^ and GFAP positive cells in the total cells (A) from middle turbinates of 7 patients. Representative photographs of middle turbinate tissue: patient 1 (B) and patient 6 (C). In C, bone was separated from lamina propria. Note that the size of samples from two patients was similar, but the yields of OECs were very different.

Since Schwann cells have p75^NTR^ expression [15], single labeling of p75^NTR^ can not differentiate OECs from Schwann cells. Therefore, double labeling of p75^NTR^ and GFAP was used to determine the percentage of OECs within the total cell population. There was significant variability in the percentage of OECs obtained from the mucosa of middle turbinates from the 7 patients. At the final enrichment step, the percentage of cells with double-labeling of GFAP and p75^NTR^ ranged from 9.2% to 73.2% with an average of 30.11% (Fig. 3A). The majority (>99%) of the other cells at this stage in the enrichment were fibroblasts. The purity of OECs did not depend on the size of turbinate sample obtained (Fig. 3B–C).

### Co-culture of Normal Human Neural Progenitors with Purified Fibroblasts

For the co-culture of NHNPs and fibroblasts, two different culture media were used: NPMM, the medium supporting both proliferation during free floating culture and differentiation after attachment to the PEI coated surface and GM, the medium used for culturing fibroblasts and OECs.

**Figure 4:**
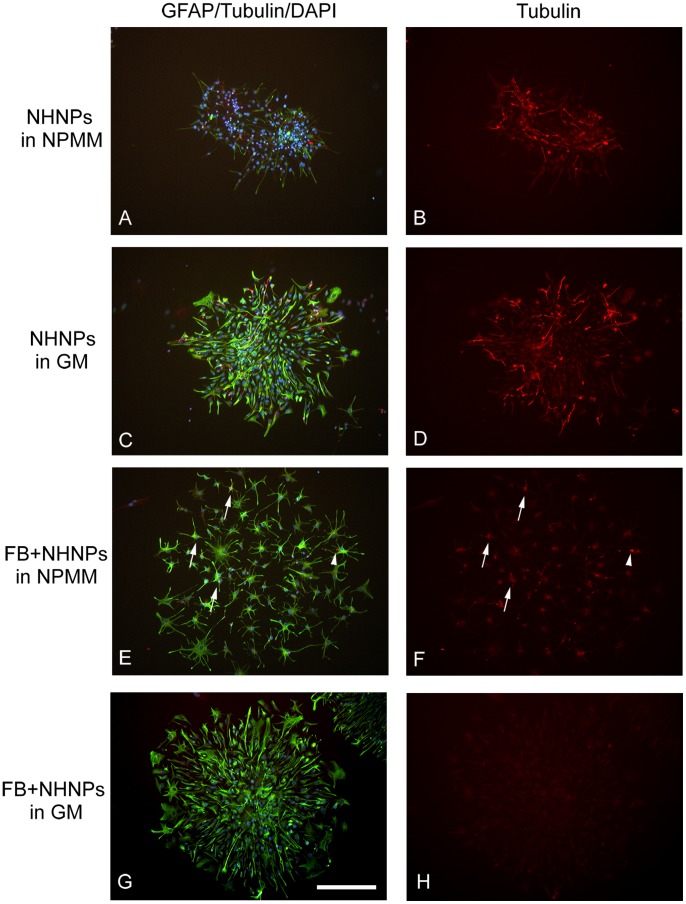
Co-culture of NHNPs with purified fibroblasts (FB) from mucosa of middle turbinate. Immunocytochemistry of GFAP (fluorescently tagged green) and β-Tubulin III (fluorescently tagged red) was performed on either NHNPs or co-culture of NHNPs with fibroblasts. Nuclei were visualized with DAPI (blue). A–B) NHNPs were plated on PEI-coated slides and cultured in NPMM. C–D) NHNPs were plated on PEI-coated slides and cultured in GM. E–F) NHNPs were plated on semi-confluent fibroblasts and co-cultured in NPMM. Arrows indicate cells with strong GFAP labeling and weak β-Tubulin III labeling. Arrow head indicates a cell with β-Tubulin III positive labeling and neural morphology. G–H) NHNPs were plated on semi-confluent fibroblasts and co-cultured in GM. Scale bars: 100 µm.

Floating spheres of NHNPs from the proliferation stage were plated on PEI-coated slides. After attachment, cells migrated out of the neural spheres and differentiated along the neuronal or glial lineages. The morphologies of these cells changed from round cells into cells with double or multiple branches. Single labeled cells with either GFAP (glial marker) or β-Tubulin III (neuronal marker) were present, indicating that NHNPs can differentiate towards either a neuronal or glial lineage (Fig. 4A, B). If NHNP spheres were cultured in GM on PEI-coated slides, the cells differentiated further into flattened cells and bipolar or multipolar cells (Fig. 4C). The colonies had denser GFAP labeling (Fig. 4C) compared to those in NPMM (Fig. 4A). However, intense β-Tubulin III single labeling was still found in many cells (Fig. 4D). Therefore, NHNPs cultured in GM or NPMM differentiated into glial or neuronal lineage cells.

NHNPs and fibroblasts were co-cultured in either NPMM or GM. Instead of seeding NHNPs on the PEI-coated slides, spheres were seeded on semi-confluent fibroblasts isolated from human lamina propria. When co-cultured in NPMM, the NHNPs migrated out extensively from the spheres and the majority of the cells presented a glial stellate morphology with intense labeling of GFAP (Fig. 4E). Some of these cells also had weakly labeled β-Tubulin III in the cytoplasm (arrows in Fig. 4E, F), indicating an intermediate differentiation stage. A few NHNPs in the colonies were single labeled with β-Tubulin III (arrowhead in Fig. 4F), indicating differentiation into the neuronal lineage. These findings demonstrated that NHNPs co-cultured with fibroblasts in NPMM preferentially differentiated into glial lineage cells. When NHNPs were co-cultured with fibroblasts in GM, they migrated out and presented as differentiated glial morphologies (Fig. 4G). The NHNPs were positively labeled with GFAP only (Fig. 4G). No β-Tubulin III labeling was seen in any of the cells (Fig. 4H). In summary, the presence of mucosal fibroblasts caused the NHNPs to preferentially differentiate along the glial lineage in two different culture media.

## Discussion

Human olfactory mucosa is located in the roof and part of the walls of the nasal cavity. The olfactory neuroepithelium extends to the upper portions of the nasal septum and onto the superior and middle turbinates. The distribution of the olfactory epithelium in adult humans is frequently disrupted with interspersed patches of respiratory epithelium [13]. Feron et al. collected 97 specimens from 6 different regions in the nasal cavity and found that the presence of olfactory epithelium ranged from 30% to 76%. Specifically 73% of specimens from the superior turbinate and 58% of specimens from the middle turbinate contained olfactory epithelium [31]. The dorsoposterior regions of the nasal septum and the superior turbinate provided the highest probability of collecting olfactory epithelium. The yield of OECs from the mucosa of septum and superior turbinate has been studied [32], [16]. Even with the improvements in surgical technique and localization of olfactory mucosa, the yield of OECs was low and highly variable, independent of the specimen size. For example, the majority of biopsies (48%) in superior turbinate resulted in OEC yields of less than 5% and only 23% with an OEC proportion of more than 50% [16]. The present study is the first to analyze the yield of OECs from the mucosa of the middle turbinate. High variabilities between individuals were also found (range from 9.2 to 73.2%). The yield was not related to the size of the biopsies. Our enrichment method was based on the different adhesion properties of each cell type and was modified from a method used to culture OECs derived from olfactory bulbs [24]. This method did not produce high purity due to the complex cell composition of the olfactory mucosa [33]. Different purification methods have been developed for elimination of fibroblasts by Thy1.1 complement lysis [34] or specific selection of OECs by p75^NTR^ antibody labeling [35], [36], [37]. Bianco *et al.* cultured OECs from human nasal mucosa of the septum and superior turbinate. OECs were purified and enriched in a serum-free medium supplemented with NT3 [15]. It was reported that 90 to 95% of the cells were GFAP or S100 positive and nearly all cells were also p75^NTR^ positive. In comparison, this study demonstrated that OECs can also be cultured from mucosa of middle turbinates, which can be collected by a less invasive procedure. However, the purification methods need to be optimized to reach higher purity for clinical usage.

OECs ensheath olfactory nerve bundles along their course through lamina propria in peripheral nervous system, across the cribriform plate and termination on the outer layer of the olfactory bulbs in the CNS [38]. OECs with different antigenic profiles and morphology led to the conclusion that there were two different subpopulations of OECs, Schwann-cell like and astrocyte-like [34]. These subpopulations of cells displayed different migratory properties *in vitro* [39]. However, further evidence showed morphological plasticity of OECs suggesting that they are a single but malleable phenotype [40]. A rapid switch (change occurred within an hour) between two phenotypes was captured in an individual OEC [41]. With our isolation procedure, two subpopulations were found in the cultures based on their antigenic and morphologic difference. Due to the low purity of OECs obtained from mucosa of middle turbinates and high variability between each specimen, co-culture of OECs with neural progenitors was not repeatable for functional distinction between the two subpopulations of OECs.

Several groups have reported the results of clinical trials of OEC autologous transplantation in SCI patients. Mackay-Sim’s group in Australia transplanted human OECs purified from nasal biopsies into the injured spinal cord of three patients with paraplegia. Biopsies from nasal septum in the superior region were collected one month before transplantation. OECs were then enriched and proliferated *in vitro* to reach high purity and large cell number. Another surgery was performed a month later to transplant OECs into the injured spinal cord. One year and three year follow-ups showed that transplantation of autologous OECs into injured spinal cord was feasible and safe up to 3 years post-implantation. Although no significant functional changes were seen in any patients, the conclusion on efficacy was deemed preliminary due to the small number of patients [8], [9]. Instead of using purified OECs, other groups used olfactory mucosa autografts from human nasal cavity. The surgeries for mucosa collection and autologous transplantation in chronic SCI patients were conducted on the same day. First, scar tissue of the lesion was removed from the spinal cord. Second, olfactory mucosa was collected and cut into small pieces and then transplanted into the lesion area to fill the cavity. Two separate sites, one in Portugal and another in India, conducted this procedure developed by Lima [10], [11], [12]. Patients showed different levels of functional improvement, such as improved motor scores and sensory neurological scores. However, there were also some adverse events reported, such as transient pain, one report of sensory decrease and one of a decreased AIS (American Spinal Injury Association Impairment Scale) grade. Despite the clinical trials, there is no agreement on whether purified OECs or tissues containing mixed cell types including OECs should be used for transplantation.

The main concerns of transplanting mucosa tissue are the unknown functions of each cell type and their interactions *in vivo*. Fibroblasts within the mucosa could be potential inhibitors of axonal regeneration due to their excreted extracellular matrix molecules [18]. In this study, the fibroblasts from nasal mucosa were stained positive for CSPG, which is a primary molecule in glial scar-mediated inhibition after CNS injuries [42], [43]. A recent paper reported that transplantation of fibroblasts or fibroblasts combined with other cell types into injured spinal cord resulted in more extensive lesion size and provoked a marked astrocytic response compared to transplantation of purified glial cell populations [44]. On the other hand, fibroblasts filled a cystic lesion in injured spinal cord [45] and when co-transplanted with neural progenitor cells, they acted as a supporting scaffold for the neural progenitor cells, which further provided a permissive environment for axonal regrowth after SCI [46]. In the same study, when rat neural progenitor cells were co-cultured with fibroblasts, the percentage of glial GFAP expressing cells significantly increased [46]. In this study, the presence of mucosal fibroblasts caused the NHNPs to differentiate into glial lineage cells, which is consistent with the previous rodent study.

Whether fibroblasts act as a supportive cell or an undesirable cell resulting in increased scar formation and inflammatory response after transplantation into CNS lesions is still controversial. Fibroblastic interaction with the donor tissue and their function after transplantation requires further investigation before translation to clinical application.
